# Study on chemotaxis and chemokinesis of bone marrow-derived mesenchymal stem cells in hydrogel-based 3D microfluidic devices

**DOI:** 10.1186/s40824-016-0070-6

**Published:** 2016-08-02

**Authors:** Dayoung Yoon, Hyerim Kim, Eojin Lee, Min Hee Park, Seok Chung, Hojeong Jeon, Cheol-Hee Ahn, Kangwon Lee

**Affiliations:** 1 Center for Biomaterials, Korea Institute of Science and Technology, Seoul, Republic of Korea; 2 Research Institute of Advanced Materials (RIAM), Department of Materials Science and Engineering, Seoul National University, Seoul, Republic of Korea; 3 Program in Nanoscience and Technology, Graduate School of Convergence Science and Technology, Seoul National University, Seoul, Republic of Korea; 4 School of Mechanical Engineering, Korea University, Seoul, Republic of Korea; 5 Advanced Institutes of Convergence Technology, Gyeonggi-do, Republic of Korea

**Keywords:** Chemotaxis, Mesenchymal stem cells, Chemokinesis, Microfluidic device

## Abstract

**Background:**

Controlling the fate of mesenchymal stems cells (MSCs) including proliferation, migration and differentiation has recently been studied by many researchers in the tissue engineering field. Especially, recruitment of stem cells to injury sites is the first and crucial step in tissue regeneration. Although significant progress has been made in the chemotactic migration of MSCs, MSC migration in three dimensional environments remains largely unknown. We developed a 3D hydrogel-based microfluidic-device to study the migration behavior of human MSCs in the presence of stromal-cell derived factor-1α (SDF-1α), interleukin 8 (IL-8) and Substance P (SP) which have been utilized as chemoattractant candidates of human mesenchymal stem cells (hMSCs).

**Results:**

We systematically investigated the chemotactic migration behaviors of hMSCs and their responses to SDF-1α, IL-8, and SP. SDF-1α was shown to be the most fascinating chemoattractant candidate among those factors at a certain time point. We also found that each chemokine showed different chemoattractant abilities according to their concentration. In the case of SP, this factor showed chemokinesis not chemotaxis. Especially at a 7–8 × 10^−8^ M concentration range, the chemokinesis ability driven by SP was further increased. The data suggest that some factors at the optimal concentration exhibit chemokinesis or chemotaxis in a 3D hydrogel-based microfluidic device.

**Conclusion:**

In this study on chemotaxis and chemokinesis of hMSCs, the system parameters such as chemokine concentration, system stability, and 2D or 3D microenvironment are critically important to obtain meaningful results.

## Background

Chemotactic behavior is a characteristic of various cell types engaging in biological processes such as inflammation, wound repair, organ development, neurite outgrowth, and tumor invasion [1]. A chemoattractant is defined as a chemical agent that induces cell migration toward itself. This agent includes members of the growth factors, cytokines and chemokines [2]. Chemotaxis in cells is the movement of cells toward or away from a chemical reagent. Attracted cells exhibit positive chemotaxis while repelled cells exhibit negative chemotaxis. While chemotaxis is a directional behavior, chemokinesis is the random movement of cells. Both endogenous and exogenous substances act as chemoattractants. Therefore, the harmony between endogenous stem cell recruitment and exogenous stem cell induction is one of the most critical issues for effective regenerative therapies in tissue engineering. Many researchers in the tissue engineering field have studied many kinds of chemokines or growth factors to recruit mesenchymal stems cells (MSCs) endogenously. MSCs have closely been involved in the process of healing, and their recruitment to the target area is crucial to enhance their therapeutic effect in the patients. The ability of MSCs to produce juxtacrine or paracrine factors is very important to induce regeneration from the endogenous stem cells. Studies have been done previously on some cytokines that affect the migration of MSCs to injury sites [3, 4]. There are some important cues that should be controlled such as the stemness of the MSCs, culture conditions, and delivery method to induce MSC migration [5]. Some chemokines and growth factors are known to promote selectively proliferation, migration and differentiation of MSCs [6, 7]. For instance, stromal cell-derived factor-1 α (SDF-1α) mediates cell migration by binding with CXC chemokine receptor-4 (CXCR4) at the site of injury [8–10]. However, most results previously have reported limitations because those are based on a two dimensional (2D) culture. It is well known that cells cultured using traditional 2D tissue culture methods are morphologically different from cells in humans or animals.

Our body has 3 dimensional (3D) structures. The cells compromising each organ interact with other cells or circumstances; thus, microenvironments affect cells significantly. 2D cell cultures are unable to perfectly mimic real cell microenvironments and cannot effectively study cell-cell and cell-extracellular matrix (ECM) interactions. In reality, all cells and tissues in vivo or in clinical condition are placed in 3D microenvironments, and some data from in vivo and clinical research done in 3D conditions show discrepancies with the data obtained from 2D in vitro conditions. Signals in 3D environments have a key role in cell differentiation, proliferation and migration of cells. A study on a 3D microenvironment is considered similar to the in vivo environment rather than a 2D culture which lacks, reduces or compromises important signaling events [11, 12]. Due to the limitations of 2D cell culture, 3D cell studies using microfluidic devices have greatly received attention enabling one to assay behaviors of stem cells in a controlled microenvironment with spatiotemporal conditions of the factors. There are some benefits such as a low volume of reagents, fast response time, consistent fluid flow on microscale dimensions of the concentration gradient in microfluidics [13, 14]. Therefore, 3D cell culture platforms are useful tools for mimicking the microenvironment of cells and tissues compared to 2D cell cultures. A number of 3D microfluidic models have been used to study the migration of neural stem cells (NSCs) [15, 16], leukocytes [17] and tumor cells [18]. Generation of a concentration gradient of cytokines or growth factors inducing single cell responses enable one to characterize the behavior of the cells quantitatively [19].

In this study, we investigated the chemotactic migration of human bone-marrow derived mesenchymal stem cells (hMSCs) with hydrogel-based microfluidic platforms. To control the conditions, we considered the composition of the hydrogels and microfluidic platform systems. Hydrogels are efficient devices to study chemokine gradient effects to quantify hMSC behaviors. The chemotaxis of hMSCs in microfluidic devices follows a stable gradient. Using a 3D microfluidic system, we studied the chemotactic migration behaviors of hMSCs and their responses to chemoattractants in a 3D microenvironment. Three candidates of chemoattractants, SDF-1α, Interleukin-8 (IL-8), and Substance P (SP) were investigated. Furthermore, we determined the optimum concentration for hMSC chemotaxis.

## Methods

### Microfluidic device fabrication

**Fig. 1:**
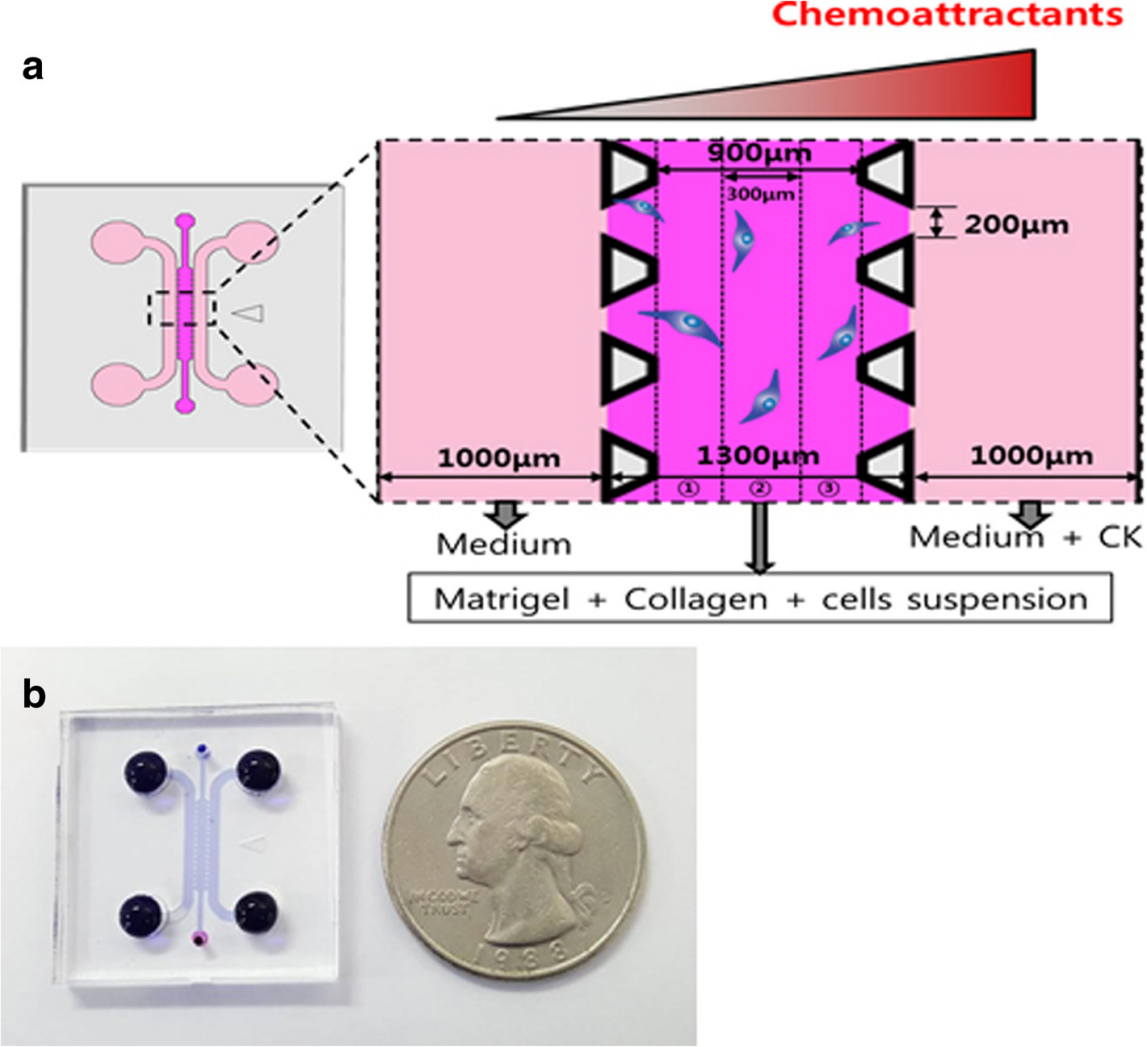
Schematic of the microfluidic device. **a** 3D microfluidic device **b** Photograph of the microfluidic device

A microfluidic device was prepared as previously described (Singapore model) [16, 20]. The microfluidic device in our study has a 1.3-mm-wide central hydrogel region flanked by two lateral media channels. The device was fabricated with polydimethylsiloxane (PDMS, Sylgard 184; Dow Corning) using soft lithography from patterned SU-8 silicon wafers. Inlet and outlet ports were created with biopsy punches, and a cover glass was bonded to the PDMS after treatment with oxygen plasma for 60 s to generate 150-um-deep microchannels. The merit of a static condition is that it is possible to investigate up to 6 different conditions including a control condition using a live cell microscope systemically and simultaneously (Carl Zeiss Axio Observer. Z1) (Fig. 1). The change in concentration gradient of the factors was tested with a standard method using 10 kDa FITC-labeled dextran. FITC-dextran mixed with the medium was injected into one channel of the device with the same concentration as the growth-factors used.

### Cell culture and cell seeding

Human Bone marrow-derived mesenchymal stem cells (hBM-MSCs) were commercially obtained from Severance Hospital. The hMSCs of passage 6 to 8 were cultured in growth medium prepared with DMEM –low glucose (Gibco) with 10 % (v/v) FBS (Welgene) and 1 % (v/v) antibiotics. Cells were maintained in culture and used up to the 7th passage. All cultures were kept in a humidified atmosphere at 37 °C and 5 % CO_2_. When needed, cells were trypsinized by standard protocols and washed in PBS. Before seeding, hMSCs at passage 7 were suspended at 2.5 × 10^5^ cells/mL in hydrogels. The hydrogels were composed of collagen (collagen type, BD Bioscience) gel (2 mg/ml) and growth-factor reduced Matrigel (GFR-Matrigel) at a 1:1 ratio (Young’s modulus: 9 kPa). The gel channel was filled with the cell suspension to complete the seeding. The gelation process occurred within 30 min in humidity boxes incubated at 37 °C and 5 % CO_2_. After gelation, the growth medium was injected into both side channels, and the gel-microfluidics were incubated in an incubator for over 24 h.

### Chemotaxis and chemokinesis assays

In the microfluidic device, hMSCs in the hydrogel were cultured overnight. After cell spreading, one of the side channels was treated with the chemokine at the relevant concentrations, and the other side of the channel was filled with blank medium. To find out the optimal concentration, each factor was screened for concentrations ranging from 10^−6^ M to 10^−10^ M. The candidates of chemokines used in this study were SDF-1α (Peprotech), IL-8 (R&D) and SP (Calbiochem).

### Cell tracking and statistical analysis

Images were taken every 10 min. for 24 h with a live cell microscope (Carl Zeiss Axio Observer. Z1) incubated at 37 °C and 5 % CO_2_. Images from the first 3 h were not analyzed until the concentration gradient of the channels was stable. In the data analysis, cells in both side sections of the central channel were not counted, and cells in section ② in Fig. 1a were analyzed. The central channel of the microfluidic device is divided by three sections, and each section has a 300 um length and a 150 um depth shown in Fig. 1a. Human MSC migration was analyzed with MTrackJ, a manual cell-tracking plugin for the NIH ImageJ software. When the cell tracking analysis was done, cells in section ② were tracked every 20 min for 10 h. Compass plots of cell tracks and angular histograms were quantified from position data using the Chemotaxis Tool plugin (ibidi, Germany). The variations in directional distance along the X-axis (ΔX) were analyzed with the Statistical Package for Social Sciences (SPSS).

## Results & discussion

### Microfluidic device identifies chemotactic migration showing stable gradients

**Fig. 2:**
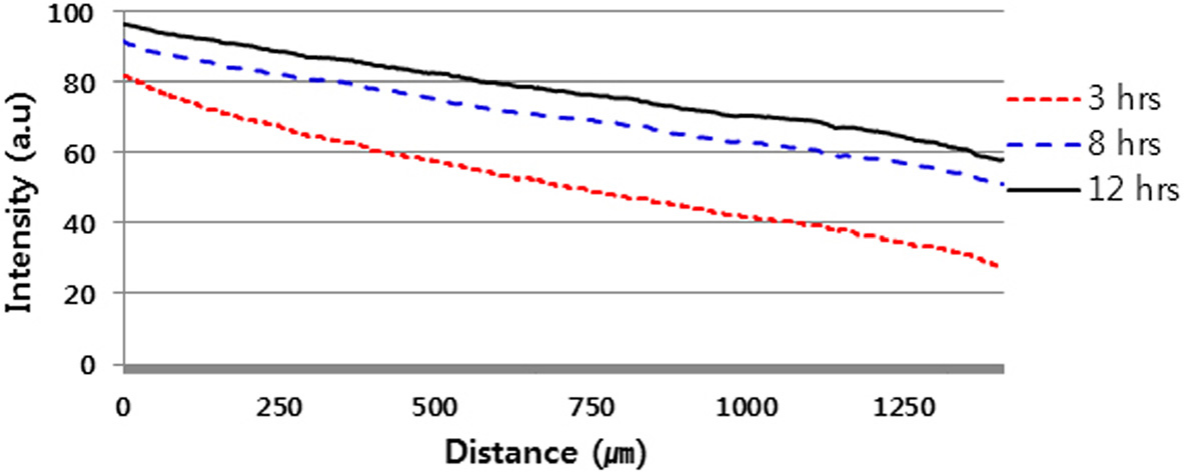
Results of the diffusion of 10 kDa FITC-dextran solution

To identify chemotactic migration, concentration gradients of chemokines must be maintained stably. We monitored the change in the concentration of the factor using FITC-dextran. Formation of stable concentration gradients in the microfluidic device was investigated with 10 kDa FITC-dextran (1 μM). After the gelation in the central channel, one of the side channels was filled with FITC-dextran solution, and the other one was filled with blank medium. Images and movies were taken every 10 min. for 13 h with a live cell microscope (Carl Zeiss Axio Observer. Z1). The data analysis was done with MATLAB. The diffusion results with the 10 kDa FITC-dextran solution showed a stable formation of the concentration gradient in Fig. 2. The slopes at 3, 8 and 12 h were 0.04, 0.03, and 0.03 a.u/μm, respectively. We observed a similar slope over 24 h, which means that our system is suitable for the test during our experimental time range. Throughout the results, stable diffusion in the microfluidic device was determined from 3 to 24 h. We systematically investigated the physical properties of the hydrogels (Matrigel, collagen only, or Matrigel/collagen mixture) to optimize the mechanical property of the hydrogel. This mechanical property of the ECM-mimicking matrix affects cell behaviors including proliferation, migration and differentiation.

### Chemokinesis of hMSCs on a chemokine gradient

To assess chemoattractant migration of hMSCs, we used the 3D microfluidic system which was designed based on the Singapore model originally developed at MIT-KU [16, 20]. One of the side channels in the microfluidic device was filled with cytokines, and the other side was filled with blank medium without cytokines. The middle channel of the device was filled with the hydrogel containing hMSC cells (Fig. 1).

**Fig. 3:**
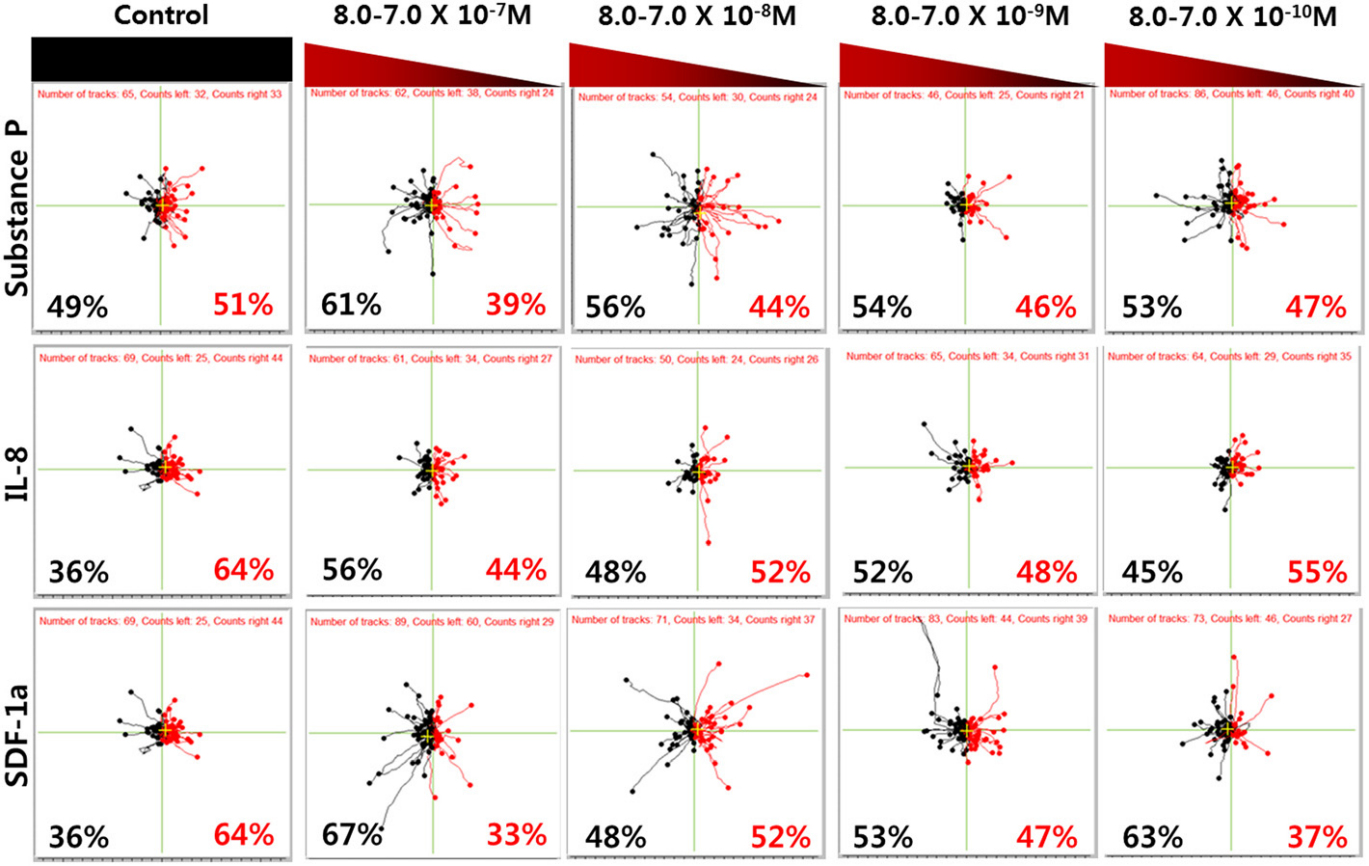
Tracking diagram of hMSCs according to the various concentration of the underlying substrates. Black pathways indicate migration of cells to the substrate, and red pathways indicate the motion in opposite direction which moves away from the substrate. The numbers in percentage indicate the ratio of migrated cells moving to each direction of tracking cells

We investigated cell movement with various concentrations of the three candidates, SP, IL-8 and SDF-1α, respectively. The control group was tested in the absence of chemokines, and the test groups used gradients of 8.0-7.0 × 10^−7^, 8.0-7.0 × 10^−8^, 8.0-7.0 × 10^−9^ and 8.0-7.0 × 10^−10^ M for the experiment (Fig. 3). SDF-1α is known as one of the potential factors inducing the chemotactic migration of mesenchymal stem cells. Compared to the control group without chemokines, the SP group showed movements of cells for concentration ranges at both 8.0-7.0 × 10^−7^ and 8.0-7.0 × 10^−8^ M. Similar to the SP group, the SDF-1α group also showed a meaningful migration of cells for concentration ranges at 8.0-7.0 × 10^−7^ and 8.0-7.0 × 10^−8^ M. However, the cells did not move in any certain direction but randomly in arbitrary directions. The distribution of cell migration in Fig. 3 was almost symmetric for both the SP and SDF-1α groups. MSCs migrate in response to an SDF-1α gradient, and the number of MSCs that migrated in the presence of SDF-1α was more than double the number of MSCs that migrated in the absence of SDF-1α which means that our result with SDF-1α is in agreement with a previous report [21]. In the results with IL-8, we did not observe any noticeable migration over the concentration ranges tested. It seems that the effect of IL-8 inducing the directional migration of hMSCs is less than the other two chemokines. This means that the hMSCs did not show any tendency of cell chemotaxis or chemokinesis compared to the control group with IL-8 in 3D conditions. A study reported previously that IL-8 secreted from kidney cancer cells induced the migration of MSCs significantly to the kidney cancer cells [22]. This discrepancy in the migration behaviors might be due to the difference in experimental conditions between the 2D in vitro (e.g., Transwell test) and the 3D study we conducted. The Transwell test commonly used in chemotaxis studies does not respond or mimic the 3D microenvironment of cells, and the data from the 2D Transwell test may give different results from the data obtained with the 3D hydrogel matrix.

**Fig. 4:**
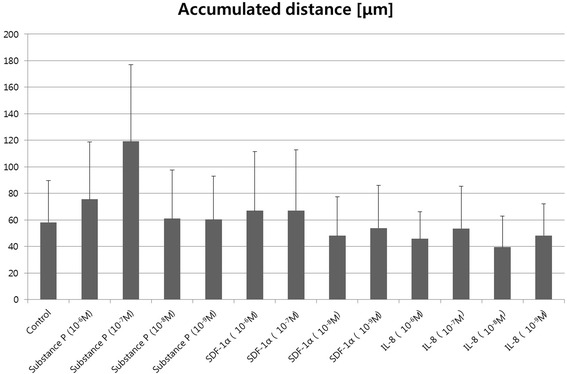
The accumulated distance of migrated hMSCs. Distance was calculated by the scalar summation of total cell movment over time

SP has also been regarded as a potential factor for the chemotactic migration of MSCs. It was reported previously that SP induces transmigration and the proliferation of bone marrow stromal cells (BMSCs) [23]. We further investigated the cumulative distance of the hMSCs which indicates the total distance of cell movement (Fig. 4). The accumulated distances were obtained from the start point of the cell migration to the endpoint of each leading edge [24]. As shown in Fig. 4, hMSCs in the 10^−7^ M SP gradient (8.0-7.0 × 10^−8^ M) had a higher value for cumulative distance compared to the control group. We confirmed that the activity of the migrated cells is in agreement with the result in Fig. 3 for SP (8.0-7.0 × 10^−8^ M). We believe that the cumulative distance in the hMSC migration test is closely related with the dynamic behavior of cells, and it means that the cells have better motility based on the higher value for the distance of the migrated MSCs.

**Fig. 5:**
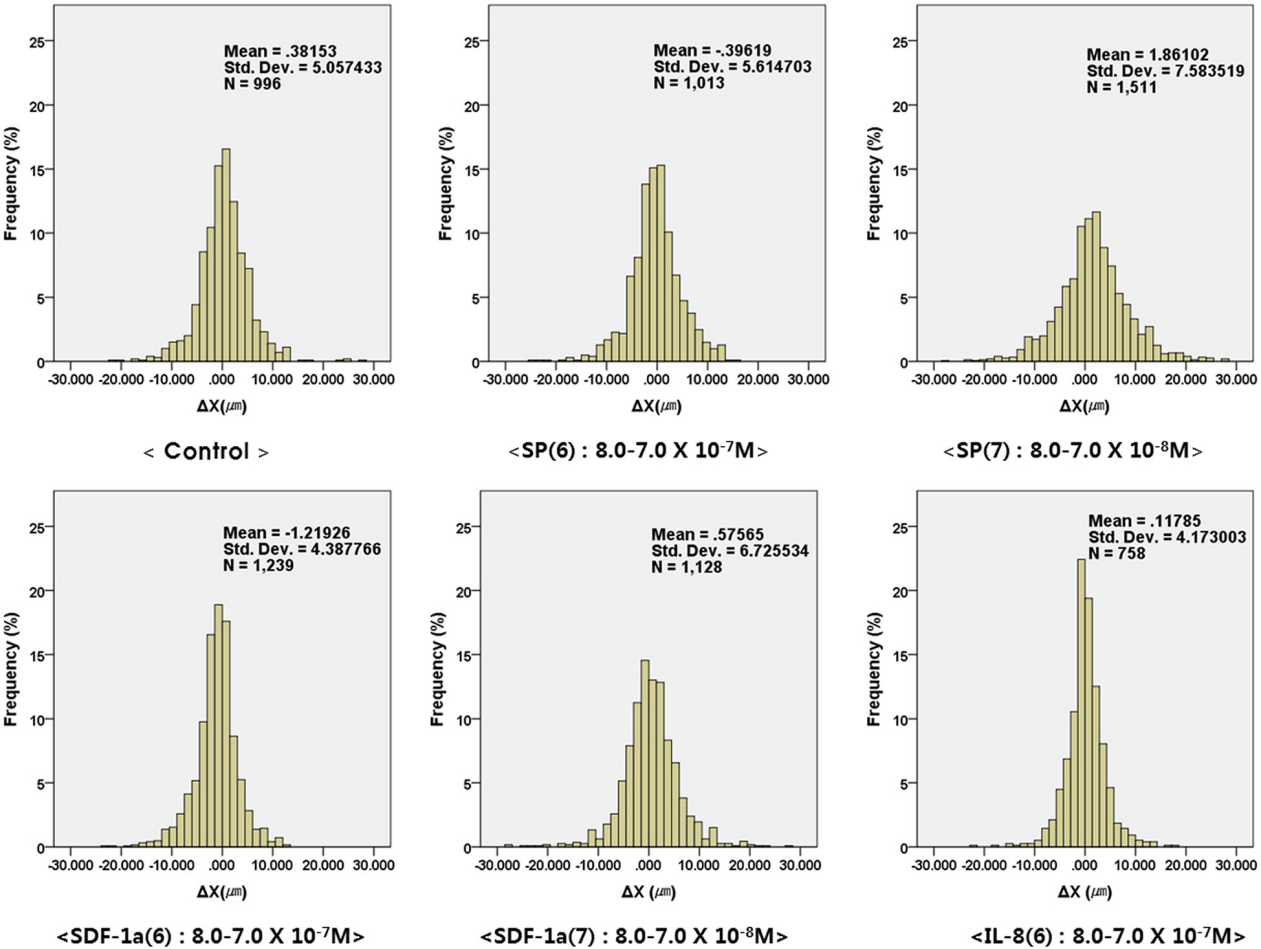
X-directional distance (ΔX) (−30 *μ*m to 30 *μ*m) analyzed by SPSS (Statistical Package for the Social Sciences). Negative signal means the movement toward high concentration, and the positive signal means the movement toward low concentration. The numbers presented on the upper right corner describe mean X-directional distance, standard deviation and total number of data

Distance analysis in the X-direction (ΔX) was done with SPSS which presented the histograms of the possibility frequency with ΔX (*μ*m) (−30 *μ*m to 30 *μ*m) (Fig. 5). Negative signals imply the migration of cells toward a direction to a higher concentration and positive signals toward a direction to a lower concentration. Moreover, tracking analysis of the migrated cells was done with the maximum concentrations of 8.0-7.0 × 10^−7^ and 8.0-7.0 × 10^−8^ M. The histogram in the case of SP (8.0-7.0 × 10^−8^ M) showed a broader distribution than that of the others. This shows that the hMSCs at this concentration range were actively migrating compared to the other groups. In addition, the histograms are uniformly distributed which means the cells did not migrate asymmetrically through the channel. Based on the result, it seems that the migration of hMSCs exhibited a chemokinesis tendency rather than a chemotaxis one.

### The chemotactic response over time

**Fig. 6:**
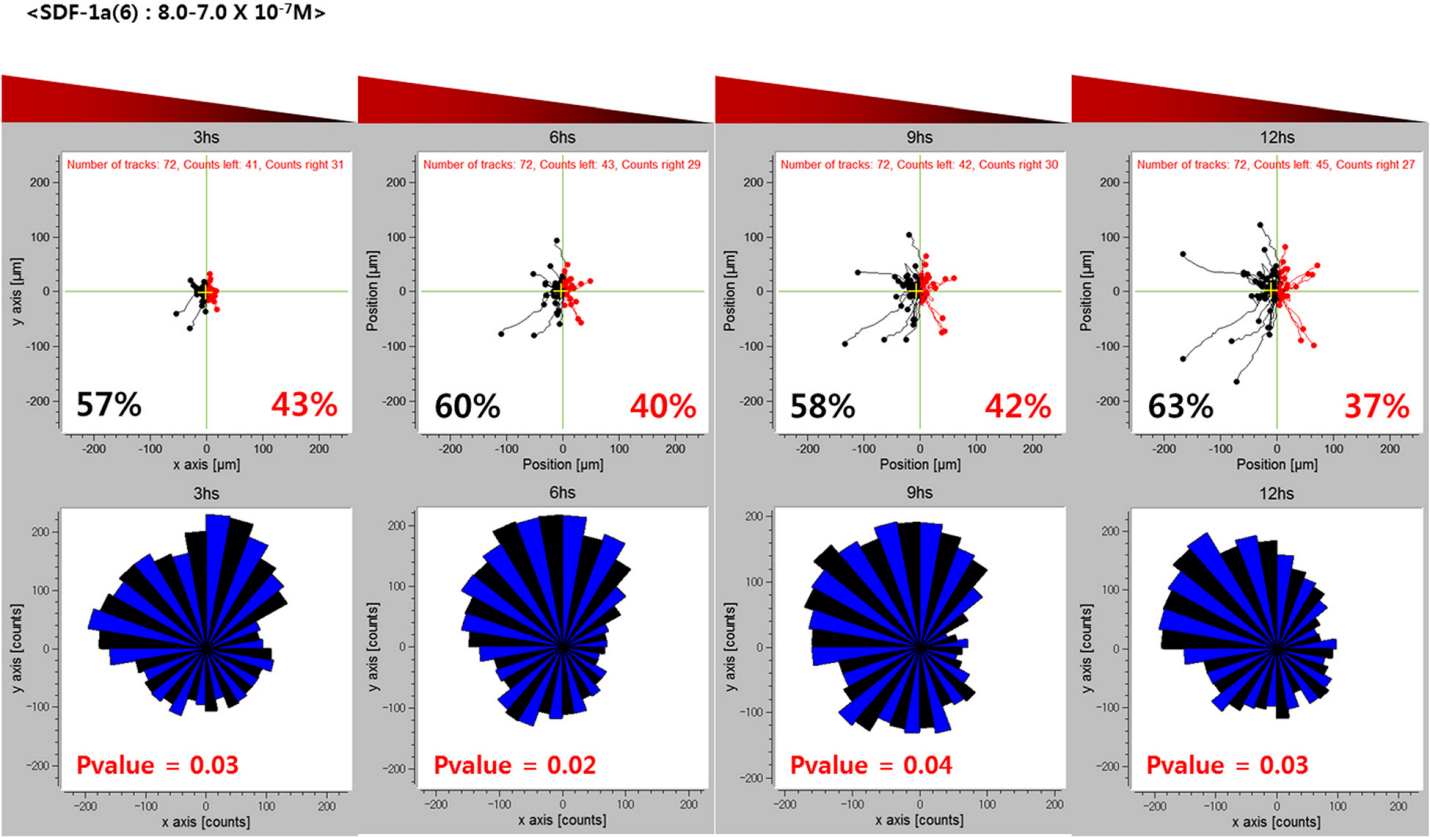
Chemotatic response on a time scale of hours. The percent of hMSCs exhibiting movement towards and away from the SDF-1α are shown in black and red fonts, respectively. The bottom plots show the corresponding rose plots, where n is the number of individual cells tracked, and p is the p-value calculated using the rayleigh test for vector data. Pvalue denote histograms with statistically significant asymmetry (*p* < 0.05)

One of the key advantages of this study using a 3D microfluidic device is that we can conduct cell migration analysis over time intervals from the recording of individual cell movements. We obtained an angular histogram by tracking cell movement for a SDF-1α concentration range of 8.0-7.0 × 10^−7^ M every 3 h for 12 h (Fig. 6). The cells migrated toward the direction of the high concentration (black pathways). The angular histogram shows an asymmetry shape at a certain time point which means that the cells showed a chemotactic migration in response to SDF-1α. This result importantly means that the chemotactic migration of hMSCs is also time-dependent which needs to be taken into consideration. Additionally, stable construction of long-term concentration gradients in microfluidic devices is essential for precise analyses of cell migration behaviors to determine if cells show chemokinesis or chemotaxis [25]. The data suggest that some factors at optimal concentrations show chemokinesis or chemotaxis in 3D hydrogel-based microfluidic devices. Many previous studies have reported on the hMSC migration behaviors induced by chemokines and growth factors in only a two dimensional (2D) culture [26–29].

## Conclusion

In this study, we systematically investigated the chemotactic migration behaviors of hMSCs and their response to SDF-1α, IL-8 and SP. SDF-1α is one of the most fascinating chemoattractant candidates at certain time points among the factors tested in this study. We also found that each chemokine exhibited a different chemoattractant ability according to its concentration. Chemokines and growth factors in previous reports induce hMSCs migration towards a high concentration which is known as chemotaxis; however, we did not observed any noticeable chemotactic behaviors in MSCs. This discrepancy between our results and the results reported by other groups might be due to the system conditions (2D vs 3D).

## Abbreviations

BMSCs, bone marrow stromal cells; CXCR4, CXC chemokine receptor-4; ECM, extracellular matrix; hBM-MSCs, human bone marrow-derived mesenchymal stem cells; hMSCs, human mesenchymal stems cells; IL-8, interleukin-8; MSC, mesenchymal stems cell; NSC, neural stem cell; SDF-1α, stromal cell-derived factor-1 α; SP, substance P; SPSS, statistical package for the social sciences
